# The involvement and application potential of exosomes in breast cancer immunotherapy

**DOI:** 10.3389/fimmu.2024.1384946

**Published:** 2024-05-21

**Authors:** Yun Wang, Qiji Ma, Tielin Wang, Jie Xing, Qirong Li, Dongxu Wang, Gang Wang

**Affiliations:** ^1^ Department of Thoracic Surgery, The Affliated Hospital to Changchun University of Chinese Medicine, Changchun, China; ^2^ Department of Breast and Thyroid Surgery, The Affliated Hospital to Changchun University of Chinese Medicine, Changchun, China; ^3^ College of Acupuncture-Moxibustion and Tuina, Changchun University of Chinese Medicine, Changchun, China; ^4^ Laboratory Animal Center, College of Animal Science, Jilin University, Changchun, China

**Keywords:** breast cancer, exosomes, immunity, tumor microenvironment, tumor immunotherapy

## Abstract

Breast cancer has a high incidence and a heightened propensity for metastasis. The absence of precise targets for effective intervention makes it imperative to devise enhanced treatment strategies. Exosomes, characterized by a lipid bilayer and ranging in size from 30 to 150 nm, can be actively released by various cells, including those in tumors. Exosomes derived from distinct subsets of immune cells have been shown to modulate the immune microenvironment within tumors and influence breast cancer progression. In addition, tumor-derived exosomes have been shown to contribute to breast cancer development and progression and may become a new target for breast cancer immunotherapy. Tumor immunotherapy has become an option for managing tumors, and exosomes have become therapeutic vectors that can be used for various pathological conditions. Edited exosomes can be used as nanoscale drug delivery systems for breast cancer therapy, contributing to the remodeling of immunosuppressive tumor microenvironments and influencing the efficacy of immunotherapy. This review discusses the regulatory role of exosomes from different cells in breast cancer and the latest applications of exosomes as nanoscale drug delivery systems and immunotherapeutic agents in breast cancer, showing the development prospects of exosomes in the clinical treatment of breast cancer.

## 1 Introduction

Despite advances in screening and treatment methods, breast cancer continues to be the predominant form of cancer for women (1). Breast cancer is the most commonly diagnosed cancer in women worldwide and the leading cause of cancer death, with an estimated 2.3 million new cancer cases and 685,000 cancer deaths in 2020. Breast cancer cases in women are expected to increase by 31% in 2040 compared to 2020 (2). Based on expression of estrogen receptor (ER), progesterone receptor (PR), and human epidermal growth factor receptor 2 (HER2), breast cancer can be categorized into the molecular subtypes of HER2-positive (ER-negative, PR-negative, and HER2-positive), luminal A (ER-positive and/or PR-positive and HER2-negative), luminal B (ER-positive and/or PR-positive and HER2-positve), and triple-negative (ER-negative, PR-negative, and HER2-negative) (3). In addition, the triple-negative breast cancer (TNBC) subtype tends to have a more aggressive clinical profile, constituting approximately 15% of all cases (4).

The current treatment for breast cancer varies according to the subtype and tumor stage. It is usually based on surgery and conventional therapy, followed by adjuvant or neoadjuvant therapy (chemotherapy, radiotherapy [RT], and immunotherapy) (5). Nevertheless, around 40% of TNBC tumors recur, with cancer metastasis being the primary factor contributing to mortality among patients with breast cancer (6). Due to clinicians’ inability to accurately predict patients’ risk of developing metastatic disease or how they will respond to treatment, up to 20% die from metastatic breast cancer (7). Given the limited overall survival rates of patients with breast cancer and the absence of universally defined molecular targets for treatment, there is a pressing need to explore and develop novel therapeutic approaches for patients with breast cancer (8).

Immunotarget therapies have become a fundamental approach in treating numerous cancers. “Hot tumors,” characterized by T cell infiltration, interferon (IFN), and elevated programmed cell death 1 ligand 1 (PD-L1) levels, show a more favorable response to immunotherapy than “cold tumors,” which lack T cells and other immune components within their tissue (9). Breast cancer is considered a “cold” tumor, and its immunotherapy and immune microenvironment still receive significant attention (10). There is an urgent need to expand the immune-targeted drugs for breast cancer and further understand the regulatory mechanism of its immune microenvironment.

The tumor microenvironment (TME) is formed by tumor stroma or mesenchyme, including immune cells, fibroblasts, extracellular matrix (ECM), blood vessels, and extracellular components (e.g., cytokines, chemokines, and exosomes) (11). The signal transduction pathway mediated by exosomes can regulate TME, which is an important way for exosomes to influence tumorigenesis and progression (12). Immunotherapy is a crucial approach in treating breast cancer, with unsatisfactory outcomes often attributed to an aberrant TME. Therefore, remodeling the TME to enhance the efficacy of breast cancer treatment and improve prognosis is greatly important (13). Enhancing the immunosuppressive microenvironment and manipulating immune cells and cytokines could contribute to optimizing antitumor therapy for breast cancer (14).

In recent years, the development and application of breast cancer nano-therapeutics have been constantly evolving to compensate for the inherent shortcomings of traditional therapies (15). Among them, exosomes have great potential to treat breast cancer because they have cell and tissue tropism, they can efficiently accumulate in tumors, and their contents or composition can be configured and modified (16). Exosomes are 30–150 nm nanoscale vesicles with phospholipid bilayer membranes secreted by eukaryotic cells (17). Endogenous exosomes can be used to enhance the delivery of drugs with low immunogenicity to tumors (18). In addition, exosomes can impact numerous vital cellular processes and the properties of breast cancer cells, including their proliferation, migration, invasion, and metastasis (19, 20). The RNA and protein contents of exosomes may be related to the reprogramming of receptor cells, such as immune cells, and have the potential for immune regulation (21). Exosomes are involved in antigen presentation and immune activation during cell communication, which may act as a medium for the interaction between cancer and immune cells. They may also be involved in avoiding immune surveillance during tumor development (22). Therefore, immune microenvironment-associated exosomes are promising therapeutic targets for early cancer diagnosis, immune regulation, and drug therapy. This review summarizes recent advancements in the research on exosomes for treating breast cancer, focusing on their regulatory impact within the tumor immune microenvironment, particularly those created by tumor and immune cells. It also discusses the potential of exosomes as therapeutic vectors to improve the efficacy of conventional cancer immunotherapy and novel therapies resulting from combining biomaterials and genetic engineering.

## 2 Exosomes influence tumor development

### 2.1 Exosomes from tumor cells promote breast cancer metastasis

**Figure 1:**
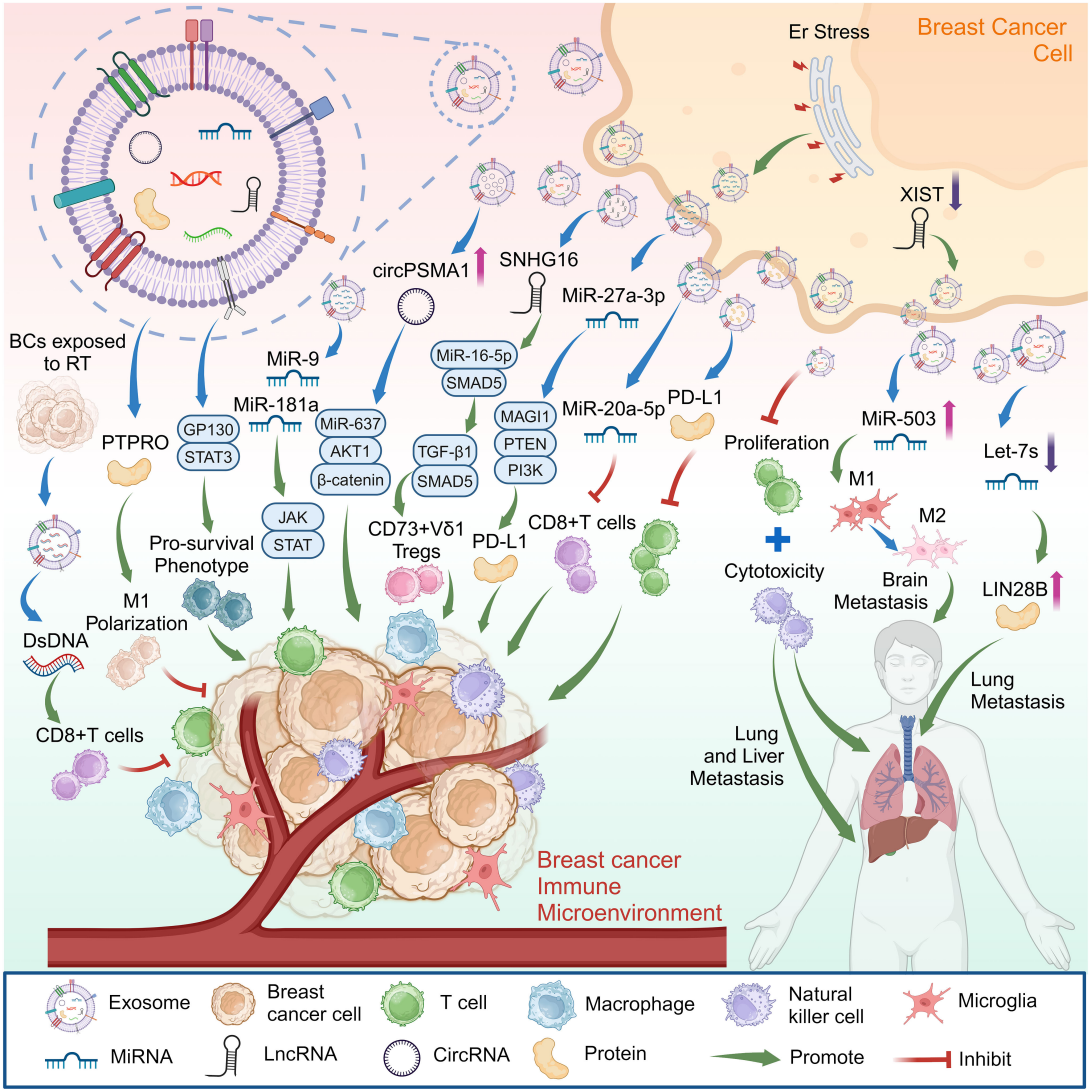
Exosomes from breast cancer cells can affect breast cancer progression by promoting cancer metastasis, regulating the immune microenvironment of breast cancer. Created with BioRender.com.

Numerous studies have indicated that exosomes derived from cancer cells can guide immune cells to enhance tumor characteristics (**Figure 1**). These exosomes are crucial in promoting various aspects of tumor progression, such as stimulating tumorigenesis, infiltrating surrounding tissues, facilitating angiogenesis, inducing the formation of pre-metastatic niches, and promoting metastatic spread (23, 24).

Metastasis is the phenomenon where cancer cells disseminate to distant locations, and exosomes are recognized as essential mediators in forming the niches preceding metastasis (25). Experiments conducted with mouse models of breast cancer and samples from patients with cancer have indicated that cytokines including C-C motif chemokine ligand 2 (CCL2), interleukin-6 (IL-6), chemokine (C-X-C motif) ligand 1 (CXCL1), and granulocyte-macrophage colony-stimulating factor (GM-CSF) interact with cancer-derived exosomes in the TME by binding to the glycosaminoglycan side chains of their proteoglycans. This interaction influences the accumulation of organ-specific exosomes, contributing to the promotion of cancer metastasis (26). Besides, some studies have indicated increased circular RNA circPSMA1 levels in exosomes originating from TNBC cells. Exosomal circPSMA1 was found to enhance the processes of tumorigenesis, metastasis, and migration in TNBC by modulating the microRNA (miRNA)-637/AKT serine/threonine kinase 1 (AKT1)/β-catenin (CTNNB1; cyclin D1 [CCND1]) axis (27).

Additionally, research findings indicate that exosomes derived from breast cancer contribute to the development of an immunosuppressive microenvironment, facilitating breast cancer metastasis and colonization. Moreover, breast cancer exosomes can directly inhibit T cell proliferation and suppress the cytotoxicity of natural killer (NK) cells, potentially impeding the anti-cancer immune response in organs before metastasis (28). Furthermore, the pluripotent factor lin-28 homolog B (*LIN28B*) is highly expressed in breast tumors, and studies have shown that low-let-7s exosomes released by tumors are a prerequisite for LIN28B-induced immunosuppression. LIN28B promotes the lung metastasis of breast cancer by establishing an immunosuppressive pre-metastatic ecological niche, enabling neutrophil recruitment and pro-tumor (N2) transformation, and promoting cancer progression (29).

Additionally, approximately 30% of patients with metastatic breast cancer eventually develop brain metastasis, and tumor cells adapt to the brain microenvironment primarily by reprogramming cells within the brain metastasis niche, which is composed of astrocytes, microglia, and various immune cells (30). Microglia are the primary innate immune cells in the brain and are a vital component of the TME in brain metastasis. Like macrophages, activated microglia have both tumor-suppressing (M1) and tumor-promoting (M2) effects (31). Studies have shown that the long non-coding RNA (lncRNA) X inactive specific transcript (XIST) plays a crucial role in the brain metastasis of patients with breast cancer by affecting tumor cells and TME. Deleting *XIST* also increases the secretion of exosomal miRNA-503. It can potentially induce the M1-M2 polarization of microglia and suppress the proliferation of T cells, making it a promising target for effectively treating brain metastasis in breast cancer (32).

### 2.2 Exosomes from tumor cells regulate PD-1/PD-L1

PD-L1 is an immune surface protein that inhibits the antitumor activity of T cells by binding to the programmed cell death-1 (PD-1) receptor, effectively shielding tumors from immune surveillance (33). Research has shown that the PD-L1 carried in exosomes originating from human breast cancer cells plays a defensive role by actively impeding the ability of T cells to eliminate breast cancer cells, facilitating tumor growth (34).

TME stress that disrupts protein homeostasis produces endoplasmic reticulum (ER) stress. ER stress promotes exosome secretion and increased exosomal miR-27a-3p, which can promote immune escape by upregulating *PD-L1* via the membrane-associated guanylate kinase, WW and PDZ domain-containing (MAGI1)/phosphatase and tensin homolog (PTEN)/phosphoinositide-3 kinase (PI3K) axis in breast cancer (35). Furthermore, combining PD-1 monoclonal antibodies with chemotherapy is currently widely used to treat patients with TNBC. However, clinical experience shows that most patients will develop resistance after prolonged treatment (36). Some studies have found that circulating miR-20a-5p released by TNBC cells through exosomes promotes cancer cell growth by inducing cluster of differentiation 8 (CD8^+^) T cell dysfunction, leading to immunosuppression. Modulating miR-20a-5p could emerge as a novel approach to overcome the resistance of TNBC to anti-PD-1 immunotherapy (37).

### 2.3 Exosomes from tumor cells regulate immune cells in the immune microenvironment

Gamma delta (γδ) T cells play an immunosuppressive role in various solid malignant tumors. They are the main component of breast cancer tumor-infiltrating lymphocytes, which are closely associated with poor pathological features and prognosis (38). Breast cancer cells have been shown to promote SMAD family member 5 (*SMAD5*) expression in γδT1 cells by transferring exosome lncRNA small nucleolar RNA host gene 16 (SNHG16), which acts as a competing endogenous RNA by sponging miR-16–5p, thereby enhancing the transforming growth factor (TGF)-β1/SMAD5 pathway and upregulating 5’-nucleotidase ecto (NT5E/CD73) levels. These results suggest that targeting CD73^+^ γδT1 cells or blocking tumor cell-derived exosomes may be potential strategies for TNBC therapy (10).

Additional research has demonstrated that miR-9 and miR-181a, originating from exosomes derived from breast cancer cells, activate the Janus kinase (JAK)/signal transducer and activator of transcription (STAT) signaling pathway. Through their respective targeting of suppressor of cytokine signaling 3 (SOCS3) and protein inhibitor of activated STAT 3 (PIAS3), these miRNAs help induce the proliferation of early myeloid suppressor cells within breast cancer and exert a potent inhibitory influence on T cell immunity in both mice and humans (39). Moreover, bone marrow-derived macrophages (BMDMs) can internalize exosomes originating from tumors. Studies have shown that exosomes derived from breast cancer cells induce a change in the macrophage phenotype through the IL-6 receptor beta (glycoprotein 130, GP130)/STAT3 signaling pathway, helping to establish a tumor-promoting TME (40).

There are crucial interactions between the cancer cells that were killed and molecules within the immune system, of which damage-associated molecular patterns (DAMPs) are frequently recognized as intracellular molecules able to play a pivotal role in immune stimulation, inducing a shift from a cold to a hot TME (41). Exosomal contents will change under diverse treatment conditions, stimulating the immune system through DAMPs. Therefore, studies have examined stimulating breast cancer cells through photothermal therapy to make them secrete exosomal DAMPs. They have found that exosomes released by dying cancer cells stimulate the immune system and inhibit tumor growth more effectively (9).

Moreover, RT leads to the accumulation of cytoplasmic double-stranded DNA (dsDNA) in cancer cells, which is one of the critical signals for the spontaneous activation of anti-immunogenic tumors by antitumor T cells and the initiation of T cells. The dsDNA activates type I IFN (IFN-I) through the cyclic GMP-AMP synthase (CGAS)/stimulator of interferon response cGAMP interactor 1 (STING1) pathway and induces multiple IFN-stimulating genes (42). Tumor-derived exosomes released by radiation-irradiated breast cancer cells were found to carry dsDNA that could induce dendritic cells (DCs) to produce IFN-I in a STING-dependent manner. Moreover, exosomal dsDNA was regulated by triple repair exonuclease 1 (*TREX1*) expression in parental cells, which induced tumor-specific CD8^+^ T cell responses and produced protective antitumor immunity in mice (43).

In addition, the protein tyrosine phosphatase receptor type O (PTPRO) is a tumor suppressor. Studies have shown that PTPRO in exosomes derived from tumor cells promoted the polarization of M1-like macrophages and modulated their associated functional phenotype. Furthermore, exosomal PTPRO from breast tumor cells inhibited the invasion and migration of breast cancer cells while concurrently deactivating STAT signaling in macrophages (44).

### 2.4 Exosomes from immune cells regulate the immune microenvironment

Immune cells can recognize neoantigens as well as endogenous and exogenous ligands and may be used for chronic inflammation and immune monitoring. Immune cells in normal breast tissue are mainly located in the epithelial components of the ductal lobules of the breast. Progression to breast cancer is characterized by increased infiltration of immune cells in the tumor parenchyma and stroma (45). Immune cell infiltration in breast cancer consists of a variety of cell subtypes, including T-cell cluster of differentiation 3 (CD3^+^), CD4^+^ and CD8^+^ cells, B cells, monocytes/macrophages, DCs, and NK cells (46).

**Figure 2:**
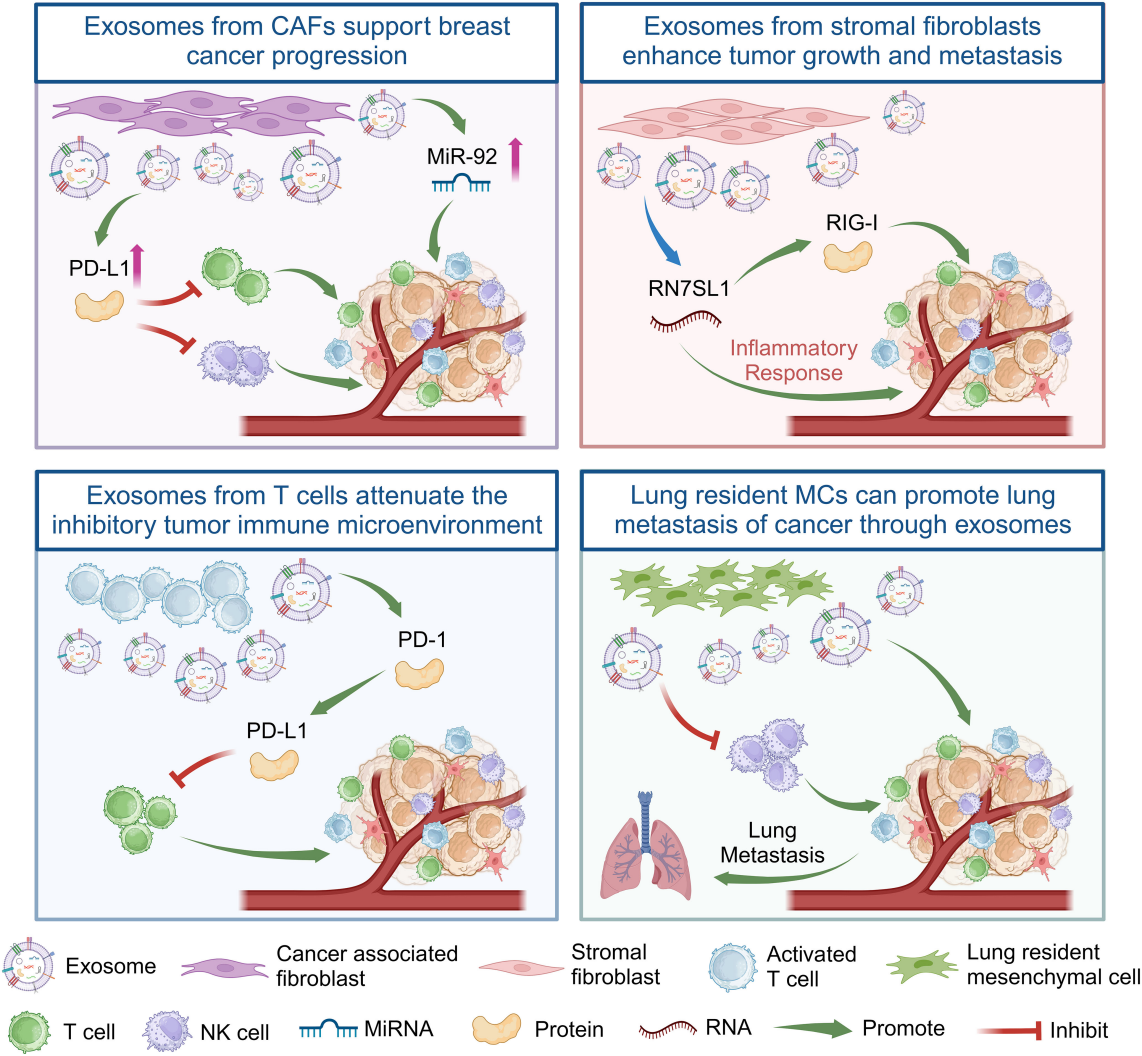
Exosomes from CAFs, stromal fibroblasts, T cells, and lung resident MCs regulate the immune response of T cells or NK cells in the immune microenvironment of breast cancer tumors by delivering non-coding RNA or proteins, thereby promoting the growth and metastasis of breast cancer. Created with BioRender.com.

The breast cancer TME comprises not only tumor cells but also stromal cells, including different subpopulations of immune cells (47), exosomes from immune cells play an important role in the regulation of breast cancer progression (**Figure 2**). Cancer-associated fibroblasts (CAFs) are crucial contributors to the breast cancer TME. Their significant role lies in aiding breast cancer progression while inducing cancer cell migration and proliferation (48). Some studies have found that *miR-92* expression significantly increases in breast cancer cells after treatment with CAF-derived exosomes. CAF-derived exosomes can significantly promote *PD-L1* expression in breast cancer cells, inducing T cell apoptosis and impairing NK cell function (49). Additionally, PD-1 can be secreted as exosomes outside activated T cells and interact with the cell surface or PD-L1 remotely to weaken the inhibitory tumor immune microenvironment by attenuating the PD-L1-induced inhibition of tumor-specific cytotoxic T cell activity (50).

In addition, the dynamic interplay between cancer and stromal cells within the TME is a notable aspect of regulated breast cancer (51). RNA component of signal recognition particle 7SL1 (RN7SL1), an endogenous lncRNA typically shielded by the RNA-binding signal recognition particles 9 (SRP9) and 14 (SRP14), is exposed within exosomes released by stromal fibroblasts. Following the transfer of these exosomes to immune cells, the unshielded RN7SL1 induces an inflammatory response. Upon breast cancer cell metastasis, the unshielded RN7SL1 activates the pattern recognition receptor retinoic acid-inducible gene I (RIGI), enhancing tumor growth, metastasis, and resistance to treatment (52). Furthermore, the environment of distant organs can potentially facilitate the metastasis of primary tumors (6). Research indicates that mesenchymal cells (MCs) in the lungs transport lipids to tumor and NK cells via exosome-like vesicles. This process results in enhanced tumor cell survival and proliferation, heightened NK cell dysfunction, the modulation of tumor cells and antitumor immunity, and ultimately promotes the lung metastasis of breast cancer (53). In summary, exosomes from different cells play an important role in the progression of breast cancer (**Table 1**).

**Table 1:** Mechanisms by which exosomes influence the development of breast cancer.

Exosome Source	Mechanism	Effect	Research object	Reference
Breast cancer cells	Microenvironmental cytokines such as CCL2 modify exosomes by binding to glycosaminoglycan side chains on the surface of proteoglycans	Exosomes accumulate in specific cell subpopulations and organs to promote breast cancer metastasis	Mouse and patients of breast cancer	(26)
Breast cancer cells	Exosomal circPSMA1 acts as a tumor promoter through the circPSMA1/miR-637/Akt1-β-catenin regulatory axis	Promotes TNBC tumorigenesis, metastasis and immunosuppression	Mouse and patients of breast cancer	(27)
Breast cancer cells	Exosomes inhibit T cell proliferation and NK cell cytotoxicity	Inhibit anticancer immune response in pre-metastatic organs	Mouse	(28)
Breast cancer cells	Exosomal Lin28B induces neutrophil recruitment and N2 transformation	Promotes lung metastasis of breast cancer by establish an immunosuppressive pre-metastatic niche	Mouse	(29)
Breast cancer cells	Loss of XIST increases the secretion of exosomal miRNA-503 and promotes M1-M2 polarization of microglia	Inhibits T cell proliferation and promotes the growth of breast cancer tumors and brain metastasis	Mouse	(32)
Breast cancer cells	Exosomal PD-L1 binds to PD-1	Inhibits T cell activation and regulates immune surveillance in the tumor microenvironment	Cells	(34)
Breast cancer cells	Exosomal miR-27a-3p upregates PD-L1 via the MAGI2/PTEN/PI3K axis in breast cancer	Promotes immune escape of breast cancer cells	Cells and patients of breast cancer	(35)
Breast cancer cells	Exosomal circulating miR-20a-5p induces dysfunction of CD8^+^ T cells	Promotes cancer cell growth and leads to resistance to PD-1 therapy	Mouse	(37)
Breast cancer cells	Exosomal SNHG16/miR-16–5p/SMAD5 regulatory axis enhances TGF-β1/SMAD5 pathway activation	CD73 expression in γδT1 cells was induced and immunosuppressive function was performed	Cells and patients of breast cancer	(10)
Breast cancer cells	Exosomal miR-9 and miR-181a activate the JAK/STAT signaling pathway by targeting SOCS3 and PIAS3, respectively	Promotes the expansion of eMDSCs, which in turn promotes breast cancer tumor growth and immune escape	Mouse	(39)
Breast cancer cells	Exosomes alter the phenotype of macrophages through the gp130/STAT3 signaling pathway	Induce IL-6 secretion in macrophages and promote tumor cell survival phenotype	Mouse	(40)
Breast cancer cells after photothermal treatment	Photothermal treatment increased the high DAMPs secreted by exosomes and the permeability of T cells to tumors	Produces immune stimulation and inhibits tumor growth	Mouse	(9)
Breast cancer cells after RT	Exosomes transfer dsDNA to DCs and activate IFN-I	Induce tumor-specific CD8^+^ T cell response and inhibits tumor development	Mouse	(43)
Breast cancer cells	Exosomal PTPRO regulates macrophage polarization	Inhibits the invasion and migration of breast cancer	Cells	(44)
CAFs derived from breast cancer	MiR-92-rich exosomes induced an increase in PD-L1 expression and promoted T cell apoptosis	Promote tumor progression and impair the function of tumor-infiltrating immune cells	Mouse	(49)
Activated T cells	Exosomes secrete PD-1 and induce the internalization of PD-L1	Inhibit tumor growth and enhance anti-tumor immunity	Mouse	(50)
Stromal fibroblasts	Unshielded RN7SL1 in exosomes activates PRR RIG-I	Enhances tumor growth, metastasis and treatment resistance	Mouse and patients of breast cancer	(52)
Lipid-Laden Lung MCs	MCs transport their lipids to tumor cells and NK cells via cellular exosome-like vesicles	Promote the survival and proliferation of tumor cells and promote lung metastasis	Mouse	(53)

## 3 Exosome-based breast cancer therapy

### 3.1 Targeted membrane-engineered exosome therapy for breast cancer

DCs are the most efficient antigen-presenting cells, which are pivotal in both triggering and orchestrating innate and adaptive immunity within the TME (54). Several vaccines designed to target DCs have been formulated and assessed in clinical trials, aiming to enhance the effectiveness of cancer immunotherapy (55). A targeted, specifically engineered exosome containing Hylton alcohol (a toll-like receptor 3 [TLR3] activator) and the immunogenic cell death-inducer human neutrophil elastase (ELANE) has been developed and optimized to form an *in situ* DC vaccine for breast cancer treatment, enhancing antitumor immunity against breast cancer (56).

The reduced efficiency of activating effector T cells results from the heightened immunosuppressive conditions in the TME (57). An approach that generates hybrids of macrophages and tumor cells has been developed to exploit the homing ability of circulating cancer cells to migrate back to the primary tumor site (58). This process involves the introduction of nuclei isolated from tumor cells into activated M1-like macrophages, resulting in the creation of chimeric exosomes termed aMT-exos. The aMT-exos can enter lymph nodes and activate T cells, targeting tumor sites, effectively inhibiting tumors, and prolonging the survival of primary breast cancer mouse models (59).

TNBC cells often overexpress the epidermal growth factor receptor (EGFR), with approximately 90% of patients with TNBC showing *EGFR* overexpression (60). An endogenous exosome carrying CD3 and a monoclonal antibody specific to cancer cell-associated EGFR has been designed and generated. The obtained exosomes could redirect and activate cytotoxic T cells to cancer cells to produce cancer-killing effects, not only inducing T cells to cross-link with *EGFR*-expressing breast cancer cells but also triggering effective antitumor immunity (61).

In addition, research has led to the creation of a synthetic polyvalent antibody retargeting exosome platform tailored to breast cancer expressing *HER2*. This platform redirects and activates cytotoxic T cells to target *HER2*-expressing breast cancer cells, achieved through the expression of anti-human CD3 and anti-human HER2 antibodies on the exosomes. The exosomes showed high efficiency and specific antitumor activity (62).

**Figure 3:**
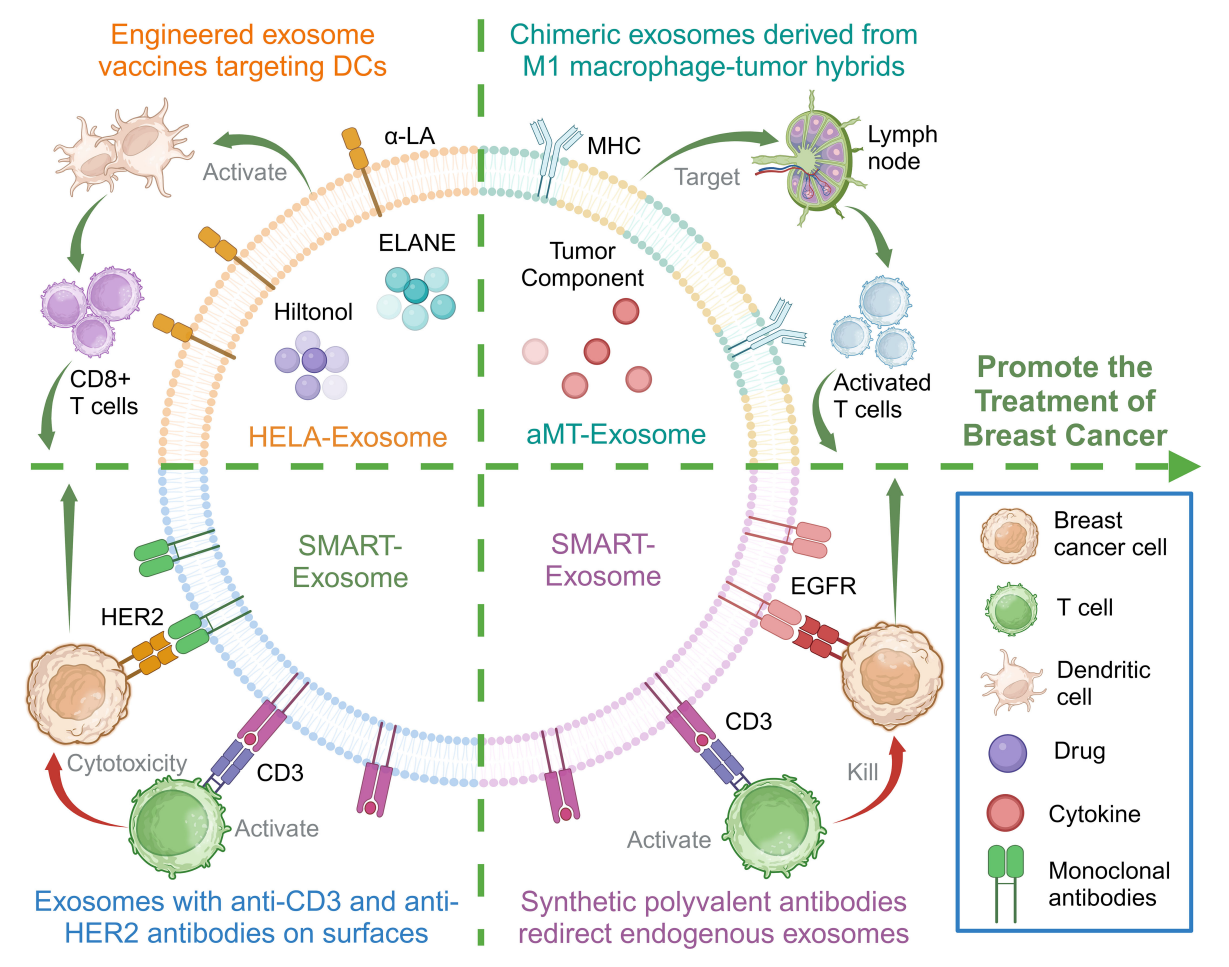
Membrane engineering on exosome surface enhances the targeting ability and immunogenicity to promote the treatment of breast cancer. Created with BioRender.com.

The aforementioned study showcases the potential preclinical viability of using natural exosomes for precise immunotherapy in treating breast cancer. Additionally, it highlights that genetically modified exosomes represent an innovative platform technology applicable to immune modulation (**Figure 3**).

### 3.2 Exosomes targeting immune checkpoints to treat breast cancer

Cancer immunotherapy, which mainly uses immune checkpoint-targeting drugs, has gained wide attention (63) (**Table 2**). There are two well-established immune checkpoint pathways: the first involves the PD-1/PD-L1 pathway, inducing immune suppression; the second involves the tumor necrosis factor receptor superfamily member 4 (TNFRSF4/GP34/CD134/OX40)/tumor necrosis factor ligand superfamily member 4 (TNFSF4/OX40L) pathway, inducing immune activation (67). Stimulating the OX40/OX40L pathway can trigger adaptive immune cells, enabling them to exert an antitumor effect (68). Due to the vital role of the OX40/OX40L pathway in immunity, increasing research and drug development have focused on it (69). OX40L M1-exos, exosomes derived from M1-like macrophages overexpressing *OX40L*, have been developed to stimulate adaptive immunity by activating the OX40/OX40L pathway. These exosomes can also reprogram M2-like tumor-associated macrophages into M1-like macrophages. The OX40L M1-exos demonstrated significant therapeutic efficacy in a mouse breast cancer model, effectively suppressing both tumor growth and metastasis (64).

**Table 2:** Exosome-associated immunotherapy for breast cancer based on immune checkpoints.

Exosome	Exosome source	Immune checkpoint	Effect	Animal model	Reference
Exosomes carrying *OX40L*	M1 macrophages	The OX40/OX40L pathway	Triggered adaptive immunity and could convert M2 tumor-associated macrophages into the M1 phenotype.	Mouse	(64)
Exosomes presenting monoclonal antibodies specific to CD3, EGFR, and immune checkpoint regulators	Expi293F cells	PD-1 and OX40L	Recruited and activated T cells and induced a robust cancer-specific immune response.	Mouse	(65)
Exosomes carrying *CD62L* and *OX40L*	DC2.4 cells	The OX40/OX40L pathway	Activated effector T cells, inhibited regulatory T cells, and inhibited tumor progression.	Mouse	(66)

Another study created genetically modified exosomes to present antibodies and immunomodulatory proteins on their surfaces. These exosome membranes presented monoclonal antibodies targeting human T cell CD3, EGFR, and the immune checkpoint regulators PD-1 and OX40L. The resulting genetically engineered multifunctional immunomodulatory exosomes effectively inhibited EGFR-positive TNBC cells and elicited potent anti-cancer immune responses *in vivo* (65).

Tumor-draining lymph node (TDLN) metastases are the most common type of breast cancer. Since the TDLN is the primary immune organ that initiates the immune response against cancer, modulating its immunosuppressive microenvironment is critical to improving the prognosis of malignant tumors (70). One study developed a genetically engineered exosome carrying selectin L (SELL/CD62L; a protein that directs lymphocytes to home to lymph nodes) and OX40L in exosome donor cells. Treating mice with breast cancer using these exosomes activated effector T cells and inhibited regulatory T cells, promoting an antitumor immune response and inhibiting tumor development (66).

### 3.3 Genetically engineered drug-loaded exosomes to treat breast cancer

There is increasing research on exosomes modification to optimize breast cancer immunotherapy (**Table 3**). The immunosuppressive TME (ITM) is often considered the leading cause of many immunotherapy failures, in which the M2 tumor-associated macrophages play a critical tumor-promoting role in the ITM (78). Iron death is a form of cell death similar to pyrodeath that can induce a robust immune response and be used to enhance immunotherapy (79). Exosomes derived from M1 macrophages loaded with ferroptosis inducer RSL3 (RSL3-exo) spontaneously homed to tumors and M2-like macrophages, polarizing them into M1-like macrophages. The RSL3-exos could also disrupt redox homeostasis and induce iron death in breast cancer tumor cells, activating an immune response and having an antitumor effect (71).

**Table 3:** Immunotherapy for breast cancer based on genetically engineered exosomes.

Exosome	Drugs	Effect	Animal model	Reference
Exosomes from M1 macrophages	Iron death inducer RSL3	Enhance the oxidative stress of tumor cells and induce ferroptosis of tumor cells	Mouse	(71)
Exosom-like nanovesicles derived from FAP genetically engineered tumor cells as a tumor vaccine	FAP	Enhance IFN-γ-induced iron death of tumor cells, activate immune cells and promote anti-tumor immune response	Mouse	(72)
Exosomes from M1 macrophages	DTX	Induction of macrophage infiltration and activation in breast cancer	Mouse	(73)
Delivery system composed of tumor exosome-liposome-PTX and gold nanorod-PEG	PTX	Promote the presentation of tumor antigen by DCs and enhance the efficacy of tumor chemotherapy	Mouse	(74)
Macrophage-derived exosomes targeting c-Met	DOX	Target tumor sites, inhibit tumor growth and induce tumor apoptosis	Mouse	(75)
Exosomes derived from NK cells	Sorafenib	Inhibit the growth of breast cancer cells and promote the apoptosis of cancer cells	Cell	(76)
Exosomes derived from breast cancer cells	MiR-155、miR-142 and let-7i	Induce DCs maturation and promote cancer inhibition	Cell	(77)

Vaccines directed towards the TME can potentially enhance antitumor efficacy by transforming the immunosuppressive TME into an immune-enhancing environment. Most exosome-based cancer vaccines have been specifically designed to target the tumor’s parenchymal cells (80). CAFs are a significant cellular constituent within the TME. They help to facilitate tumor cell invasion and contribute to the persistent activation of signals driving tumor proliferation through ECM construction and remodeling. CAFs also help promote tumor angiogenesis and participate in the intricate interplay between cancer cells and other stromal cells (81). Fibroblast activation protein-alpha (*FAP*) is overexpressed in CAFs in >90% of human tumor tissues, and exosome-like nano-vesicles derived from tumor cells with *FAP* mutations (eNVs-FAP) have been developed. This vaccine inhibited tumor growth by eliciting a potent, specific cytotoxic T lymphocyte immune response against tumor cells and *FAP*-expressing CAFs, reshaping the immunosuppressive TME in a breast cancer model (72).

One study developed a docetaxel (DTX)-M1-exosomes drug delivery system, incorporating the chemotherapy drug DTX into exosomes derived from M1 macrophages (M1-EXO) with pro-inflammatory characteristics. Its findings indicated that DTX-M1-EXO could induce the polarization of M0 macrophages into M1 macrophages, sustaining a robust and prolonged M1 activation and further stimulating macrophage infiltration and activation in breast cancer (73).

Moreover, nanomaterial-based photothermal therapy has gained considerable attention as an emerging clinical approach for treating malignant tumors. Studies have indicated that gold nanorods have a direct cytotoxic effect on cancer cells in localized tumors through thermal ablation (82). A bionic hybrid system for specific drug delivery composed of tumor-derived exosomes and liposomes was designed to overcome the targeting defects of lipid carriers. A synergistic approach was devised using gold nanorod-polyethylene glycol and exosome-liposome paclitaxel (PTX) for the targeted treatment of recurrent tumors and distant metastases in patients with advanced breast cancer. Integrating thermal ablation, adaptive antitumor immunotherapy, and targeted PTX chemotherapy collectively enhanced the therapeutic efficacy against advanced breast cancer (74).

In addition, the mesenchymal-epithelial transition factor (c-Met) is a hepatocyte growth factor tyrosine kinase receptor important in promoting epithelial-mesenchymal transformation, angiogenesis, and cell proliferation (83). Based on its overexpression on the surface of TNBC cells, c-Met can potentially become an ideal target for TNBC antitumor drug delivery (84). A c-Met targeted exosome drug delivery system was developed using engineered exosome membrane-coated nanoparticles to package Doxorubicin (DOX)-loaded polylactic acid-glycolic acid nanoparticles into macrophage-derived exosomes. The engineered exosomes demonstrated significant tumor-targeting effects *in vivo*, inhibiting tumor growth and inducing tumor cell apoptosis, and are a promising drug delivery strategy for TNBC therapy (75).

**Figure 4:**
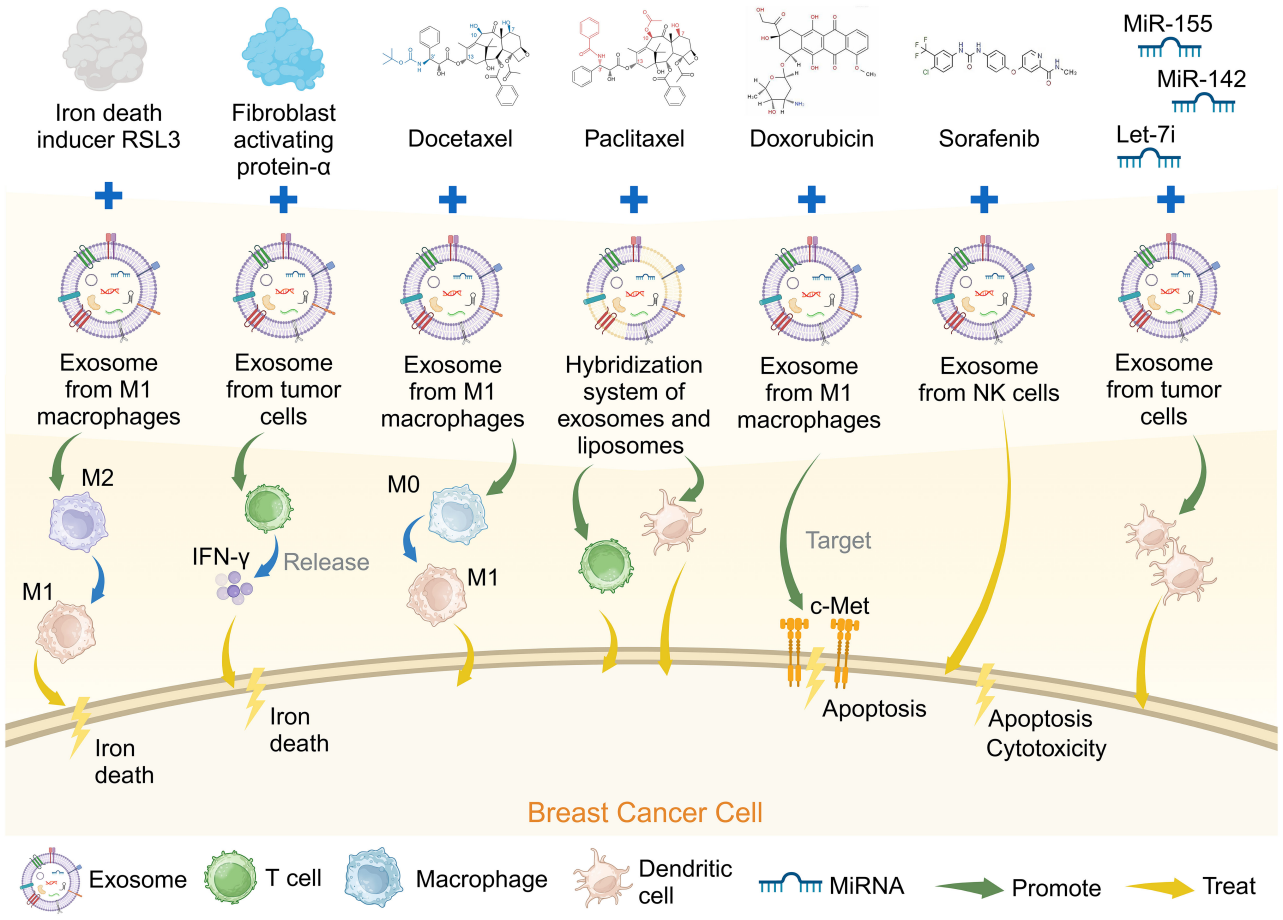
Genetically engineered exosomes loaded with breast cancer chemotherapy drugs can promote the treatment of breast cancer. Created with BioRender.com.

Other studies have loaded sorafenib into NK cell-derived exosomes to enhance apoptosis in TNBC cells, which induced significant cytotoxicity in cancer cells (76). Furthermore, several miRNAs, including miR-155, let-7i, and miR-142, are involved in DC differentiation and maturation (85). Exosomes can serve as a vector for delivering any miRNA. miR-155, miR-142, and let-7i have been used to modify exosomes derived from breast cancer cells, enhancing their immunostimulatory potential and successfully prompting DCs maturation. These findings suggest that adapted exosomes hold substantial promise as a cell-free vaccine for cancer therapy (77). Engineered exosomes with therapeutic molecules may provide new insights into immune cell-based therapies or the development of novel vaccines for breast cancer (**Figure 4**).

## 4 Discussion

The targeted therapy of immune checkpoints has shown promising results in clinical trials of various cancers. However, its efficacy in patients with breast cancer is generally unsatisfactory (86). Recent research has mainly explored potential ways to modulate the immunosuppressive TME while also inducing long-lasting antitumor activity in breast cancer. Therefore, developing appropriate tumor immune microenvironment delivery systems in preclinical studies is critical to improving the efficacy of combination therapy in patients with breast cancer. Exosomes have become key mediators of immunosuppression and immune evasion in the complex interactions between breast cancer tumors and normal cells (87). Tumor-derived exosomes may disrupt immune function and impair the efficacy of immunotherapy. In contrast, exosomes derived from immune cells may counteract these effects and promote immunotherapy, reflecting the crucial role of exosomes in regulating immune response. Therefore, they may serve as crucial therapeutic vectors targeting the tumor immune microenvironment.

The interactions between tumors and their microenvironment significantly contribute to tumor progression, metastasis, and resistance to treatment (88). Increasing evidence suggests that exosomes derived from tumor cells can systematically modulate or reprogram the TME by transferring molecules, including miRNAs, mRNAs, and proteins, from donor to recipient cells (89).

The bone, brain, liver, and lungs are the most frequent sites of metastasis in breast cancer (90). Metastasis is a significant characteristic of breast cancer, with approximately 20%–30% of patients diagnosed with early-stage breast cancer developing distant metastases. Unfortunately, complications arising from recurrent or metastatic disease contribute to the mortality of approximately 90% of patients (91).

Exosomes derived from tumors influence every phase of the invasion-metastasis cascade, and they actively contribute to the creation of pre-metastatic niches, engaging with and impeding the normal functions of immune cells, leading to the establishment of an immunosuppressive microenvironment (92). Studies have shown that interventions that interrupt exosome release or impede interactions between their contents and receptor molecules can partially reverse this remodeling of the immunosuppressive microenvironment.

In addition, immune cells such as CAFs, T cells, MCs, and NK cells can transfer exosomes to tumor cells to influence tumor fate. Since the antitumor immune response generated during immunotherapy is dynamic, the immune system must constantly rebuild its functional orientation to respond to different environmental developments. In addition, immune system elements can transmit exosomes to generate antitumor immunity or to promote tumor progression by shaping tumor immunogenicity (93). Enhancing the antitumor effect of exosomes and blocking their pro-tumor effect may be an important research target for breast cancer immunotherapy.

Immunotherapy is gaining widespread attention as a treatment option for breast cancer. Cancer immunotherapy commonly involves immune checkpoint inhibitors, cytokines, adoptive cell therapy, and cancer vaccines (94). Crucially, it is expected that exosomes originating from the TME could be highly efficient vaccines for breast cancer.

Numerous studies have shown the promising potential of exosomes as effective drug delivery carriers in anti-breast cancer treatment. These studies revealed that tumor-derived exosomes had favorable tumor-homing properties and low toxicity and immunogenicity *in vivo* (95). Modifying exosomes by genetic engineering could further enhance their targeting and therapeutic properties, expanding their clinical application.

Several studies have also designed hybrid exosomes that target immune cell activation to induce an immune response and promote the reversal of the immunosuppressive TME, which are more stable than cells and can be used after frozen storage (96). In addition, unlike molecular immunotherapies such as specific antibodies and immune checkpoint inhibitors, exosomes containing integrated immunomodulatory proteins may augment therapeutic efficacy by engaging in and regulating multiple immune checkpoint pathways. Approaches using membrane engineering to incorporate functional proteins into exosome membranes have shown enhanced stability and activity (65).

At present, most metastatic tumors are treated by systemic chemotherapy in the clinic. However, this poorly targeted treatment method can lead to drug resistance and side effects, reducing its efficacy. Exosome bioengineering methods can enhance targeted delivery to specific tissues or cells, and can be treated with smaller doses, facilitating their clinical application (97). Using exosomes as a drug delivery platform for combination therapy has shown better therapeutic effects *in vivo*, demonstrating that exosome-based targeted immune-chemotherapy is a promising candidate for treating breast cancer (74). Given the critical role of exosomes in breast cancer immunotherapy and TME regulation, it is necessary to further explore the relevant mechanisms and fully determine the safety and effectiveness of exosome-based immunotherapy, which is beneficial to developing targeted therapies for breast cancer.

## 5 Conclusions

Breast cancer is a challenging disease to cure. Therefore, new early diagnostic tools and more treatment options must be explored. The dynamic interactions between different cell types in the TME regulate various stages of breast cancer progression, and exosomes have been shown to mediate many regulatory processes. Exosomes from tumor and immune cells can promote an immunosuppressive microenvironment, tumor progression, and the immune response and inhibit tumor development. Compared to traditional nano-medical drugs, exosomes have unique advantages in drug delivery, especially genetically engineered exosomes or combining exosomes with chemotherapy drugs, which can significantly improve their targeting and therapeutic effect. Exosomes provide an opportunity for tumor immunotherapy, and factors such as the acquisition method of exosomes, biomarker detection, treatment method and drug selection, loading method of exosomes and appropriate dose for tumor treatment affect the clinical application of exosomes, which requires more extensive research and verification. In conclusion, exosomes play an essential role in breast cancer tumor immunity, modulating the immune microenvironment, underscoring the significance of investigating exosome-based immunotherapy for breast cancer.

## Author contributions

YW: Writing – original draft, Writing – review & editing. QM: Writing – review & editing. TW: Writing – review & editing. JX: Writing – review & editing. QL: Writing – original draft, Writing – review & editing. DW: Writing – review & editing. GW: Writing – review & editing.
